# Current Flavivirus Research Important for Vaccine Development

**DOI:** 10.3390/vaccines8030477

**Published:** 2020-08-27

**Authors:** Elizabeth Geerling, Tara L. Steffen, James D. Brien, Amelia K. Pinto

**Affiliations:** Department of Molecular Microbiology and Immunology, Saint Louis University, St Louis, MO 63103, USA; lizzie.geerling@slu.edu (E.G.); tara.steffen@slu.edu (T.L.S.); james.brien@health.slu.edu (J.D.B.)

The Flaviviridae family of RNA viruses includes numerous human disease-causing pathogens that largely are increasing in prevalence due to continual climate change, rising population sizes and improved ease of global travel. Escalating circulation of these emerging and re-emerging pathogens draws attention to the need for vaccines to protect against the severe diseases they cause, and this need is further exacerbated by their transmission that occurs primarily through arthropod vectors. When constructing new, efficacious vaccine candidates, several goals are targeted including safety, protective capacity, ability to confer sustained protection, induction of neutralizing antibody and protective T cell responses, as well as practicality.

Prophylactic vaccination as a means to protect against flavivirus infection has become an intense area of research in the last several decades due to the inherent nature of this family of viruses to cause explosive outbreaks [1]. However, even with the enormous prevalence and impact on the human population, there are minimal flaviviruses with approved human vaccines to date: yellow fever (YF), Japanese encephalitis virus (JEV), tick-borne encephalitis virus (TBEV) and dengue virus (DENV) [2]. This Special Issue highlights current studies that delve into research expanding the range of flavivirus candidate vaccines. These findings are essential to advance the field and further protect vulnerable populations from these disease-causing pathogens.

Although the members of the flavivirus family have been shown to be antigenically similar [3], these viruses are indeed unique enough to often warrant varying vaccines. This concept is highlighted by the four distinct DENV serotypes. DENV is widespread and endemic in greater than 125 countries and currently accounts for an estimated 100–400,000,000 infections each year, with half of the world’s population being at risk of infection [4]. As the four serotypes of DENV are distinct, infection with one serotype results in only transient protection against the other three serotypes [5], and each subsequent heterologous infection increases the risk for an infected person to develop severe dengue.

Although there is a currently approved DENV vaccine, safety and efficacy data support the need for alternative prophylactic vaccines to protect a broader population of individuals. Strides toward this goal include virus-like particles (VLPs), which are outlined in the review written by Wong et al. which summarizes VLP-based DENV vaccines currently being tested. As noted by Wong et al., many of the current VLP-based DENV vaccines lack the viral pre-Membrane (prM) protein; thus, it is believed these vaccines will circumvent the induction of cross-reactive non-neutralizing antibodies that may mediate antibody dependent enhancement. Importantly, these constructs still maintain key structural components that induce T cell and antibody responses, like envelope domain III (E DIII) against which most neutralizing antibodies are targeted [6]. Additionally, such VLP vaccines have greater safety profiles than live-attenuated vaccines, which have a higher risk of reverting to infectious virus in the elderly, a population that has been shown to be at high risk for infection and difficult to vaccinate [7,8]. The case report presented by Domingo et al. features an instance where an elderly individual received the YF vaccine prior to traveling to Brazil, then became ill and experienced septic shock and multiorgan failure. It was soon determined that he already had discernable YF antibodies a week after vaccination, in addition to high YF viral load in serum, plasma, respiratory secretions and urine [9]. Thus, the benefits of VLP vaccines include eliminating mutations to infectious virus in these critical, vulnerable populations while maintaining antigenic structural proteins in a native conformation.

Further, deeper understanding and advances in flavivirus genome structure and replication cycles have been able to aid the development of other kinds of unique DENV vaccine candidates. In this Issue, Park et al. outlines a candidate subunit vaccine based on eliciting a strong neutralizing antibody response against E DIII. This vaccine was developed to combat all four DENV serotypes by combining two subunits: partial envelope domain II and consensus envelope domain III (cEDIII) [10]. The authors found that an antibody purified from mice following immunization with this combined candidate vaccine was strongly neutralizing compared to their single subunit vaccine containing only cEDIII, with limited non-neutralizing antigen-specific antibody [10]. In addition, this combined subunit vaccine was able to reduce DENV titers in several tissues when compared to the single subunit vaccine, and conferred protection against DENV1, 2 and 4 in murine challenge models, thus showing promise as a candidate DENV subunit vaccine [10]. Similarly, Tremblay et al. highlights how the discovery that flavivirus vRNA 2′-*O*-methylation enables mimicry of cellular mRNAs has been instrumental in reverse genetics studies to improve DENV vaccine design [11]. Specifically, when mutant DENV clones are modified to encode a single point mutation in the viral nonstructural gene 5 (NS5) methyltransferase catalytic site, sensitivity to interferon treatment is enhanced, and pre-treatment with such mutant viruses protects mice and monkeys from lethal DENV infection [12]; this same approach has also proven successful in laboratory studies seeking to formulate an alternative JEV vaccine construct [13].

In addition to the previously mentioned challenges of developing flavivirus vaccines, another strong impediment to their development are practical considerations. As endemic regions of flavivirus circulation tend to be rural, access to trained medical professionals and refrigeration is often sparse. To counteract these obstacles, some vaccine constructs are aimed at achieving formulations that are biologically stable over time and do not require a medical professional to administer. In Muller et al., a tetravalent E DENV subunit vaccine was administered via a microarray patch and, when compared to traditional vaccination methods, led to enhanced neutralizing antibody titers to all four DENV serotypes potentially due to the delivery of antigens in close proximity to antigen presenting cells located beneath the skin. Inclusion of an adjuvant to enhance immunogenicity led to complete protection in mice following DENV challenge [14]. In addition to being efficacious, this form of vaccination would also be beneficial to the population since a nanopatch can be administered by someone who is not medically trained, and it does not require refrigeration.

This Special Issue also focuses its attention on Zika virus (ZIKV), a re-emerging pathogen with a history of causing epidemics: 2007 (Yap islands), 2013 (French Polynesia) and 2015 (South America) [15]. The 2015 ZIKV epidemic was rife with enhanced viral spread due to travelers, further highlighting the need for a prophylactic vaccine that can be administered to residents of ZIKV endemic areas, as well as to travelers. Similar to DENV vaccination efforts, many trial ZIKV vaccines are formulated to elicit strongly neutralizing antibodies, with several purified inactivated, live-attenuated and DNA-based vaccines correlating with protection in mice, rhesus macaque animal models and phase I human clinical trials [16,17,18]. In a study presented by Frumence et al., a chimeric ZIKV vaccine clone was generated where the glycan loop of the viral E protein of African strain MR766 was modified to encode three counterpart E glycan loop amino acids of the epidemic strain, BeH819015, formulating a chimeric clone referred to as ZIKVBeHMR-2. Following these substitutions and subsequent vaccination with ZIKVBeHMR-2, neutralizing ZIKV antibodies were rapidly detected and effective at combatting MR766 challenge in mice [19].

In addition to the need for development and maintenance of strongly neutralizing antibodies following vaccination, cellular responses have also proven to be essential in combatting ZIKV, as it has been shown that CD4 and CD8+ T cells are critical in reducing viral load and mortality in mouse models of ZIKV infection [20,21,22]. To this end, the review written by Wong et al. highlights pre-Membrane:Envelope (prME) and Capsid:pre-Membrane:Envelope (CprME) VLP-based vaccines that elicit T cell responses and stronger neutralizing antibody responses than other DNA and formalin inactivated preparations [23,24]. As previously mentioned, VLP-based vaccines have proven to be safer than live-attenuated formulations, which will be key in ZIKV vaccine development since pregnant women are classified as one of the most vulnerable populations. Nonetheless, in 2018, a live-attenuated ZIKV vaccine that was protective in animal challenge models was tested in a phase I clinical trial with support of the National Institutes of Health. The possibility of reversion that comes with a live attenuated vaccine denotes the need for a metric to examine mutation rates of such vaccines. In the study presented by Collins et al., next generation sequencing (NGS) was utilized to measure genetic diversity of live attenuated candidate ZIKV vaccines and revealed that viral adaptation occurred throughout cellular passages [25]. Thus, this study highlights a role for NGS in studying the genetic stability of live attenuated vaccines.

Another article, co-authored by our group, also focused on ZIKV vaccine development, highlighting another promising vaccine design avenue: adenovirus-vectors. These vectors have increased in popularity over the last several years as vaccine platforms primarily due to excellent safety profiles, the ability to be grown to high titers in cell culture, capacity to induce strong inflammatory immune response, etc. [26]. Steffen et al. developed an Early region 1A/Early region 3 (E1A/E3)-deleted adenovirus-vector ZIKV vaccine that included an amino acid mutation to improve prM-E polypeptide processing, in addition to including both B and T cell epitopes. Further highlighting the advantages of vaccines that elicit both humoral and cell-mediated immune responses, we showed that the vaccine construct was both immunogenic and efficacious in producing ZIKV-specific CD4+, CD8+ and antibody responses that were protective in a murine ZIKV challenge model [27]. Thus, this study provided strong evidence that an adenovirus-vector vaccine against ZIKV has the ability to induce a potent immune response against this re-emerging pathogen.

As this Special Issue highlights, developing vaccines for numerous members of the Flaviviridae family has proven to be challenging for a variety of reasons, including virus interplay with the immune system, a concept highlighted in the review written by Tremblay et al. [11]. One vaccine approach which would alleviate the concerns of flavivirus immune suppression on vaccine efficacy was highlighted by Wang et al. [28], who sought to focus vaccine efforts on the vector rather than the pathogen. Since many flaviviruses are often transmitted by the same species of arthropod vectors, some research efforts have focused on generating vaccines against antigens produced by the transmitting vector. Of particular note, it has been shown that an *Aedes aegypti* salivary gland protein, AgBR1, shapes innate immune responses in murine models following mosquito bites [29]. Based on this discovery, Wang et al. developed an AgBR1 adjuvanted active immunization strategy in mice that led to the delay of lethal mosquito-transmitted ZIKV infection [28]. This paper introduces an interesting concept where immunization with a mosquito-derived protein could protect populations from numerous mosquito-spread pathogens by targeting the innate immune response.

Hand in hand with vaccine design, methods of evaluation for vaccine efficacy are also under intense research. In a study by Frumence et al., a flow cytometry-based neutralization test (FNT) was employed as an alternative to the conventional plaque-reduction neutralization test (PRNT) as a means to enable a high throughput screening strategy that does not rely on methods requiring cell lines capable of fostering plaque formation. Through the development of a GFP reporter of ZIKV, this study highlights the ability to measure ZIKV neutralizing antibodies via flow cytometry with the antibody titers detected being equivalent to those done by PRNTs in tandem [30]. Another promising way to evaluate and enhance vaccine efficacy is highlighted in the study presented by Salat et al. where mass spectrometry was used to analyze the content of two TBEV vaccines. Upon confirming the presence of nonstructural protein 1 (NS1) in one vaccine tested, it appeared to be highly immunogenic when administered to mice and was able to enhance their survival following lethal virus challenge [31]. Thus, this study highlights another viral protein, NS1, in addition to E that can enhance the protective capacity of TBEV vaccines [31].

Overall, this Special Issue highlights numerous studies, reviews and a case report that outline current research being done to further flavivirus vaccine development. These studies address both the challenges of developing flavivirus vaccines and the diverse and novel approaches to vaccine development that are currently underway. In addition, this Special Issue also features work being done to improve pre-existing vaccines, as well as techniques to streamline the process of determining vaccine efficacy while confirming safety. While the rates of flavivirus infections continue to rise globally, research efforts like the ones outlined in this Issue continually increase our knowledge of flaviviruses, thus informing the development of novel vaccines which will meet the growing need for protection against these pathogens.

## References

[1] Holbrook M.R. (2017). Historical Perspectives on Flavivirus Research. Viruses.

[2] Collins M.H., Metz S.W. (2017). Progress and Works in Progress: Update on Flavivirus Vaccine Development. Clin. Ther..

[3] Hassert M., Brien J.D., Pinto A.K. (2019). Mouse Models of Heterologous Flavivirus Immunity: A Role for Cross-Reactive T Cells. Front. Immunol..

[4] World Health Organization (2020). Dengue and Severe Dengue.

[5] Katzelnick L.C., Gresh L., Halloran M.E., Mercado J.C., Kuan G., Gordon A., Balmaseda A., Harris E. (2017). Antibody-dependent enhancement of severe dengue disease in humans. Science.

[6] Wong S.H., Jassey A., Wang J.Y., Wang W.-C., Liu C.-H., Lin L.-T. (2019). Virus-Like Particle Systems for Vaccine Development against Viruses in the Flaviviridae Family. Vaccines.

[7] Brien J.D., Uhrlaub J.L., Hirsch A., Wiley C.A., Nikolich-Žugich J. (2009). Key role of T cell defects in age-related vulnerability to West. Nile virus. J. Exp. Med..

[8] Pinto A.K., Richner J.M., Poore E.A., Patil P.P., Amanna I.J., Slifka M.K., Diamond M. (2013). A hydrogen peroxide-inactivated virus vaccine elicits humoral and cellular immunity and protects against lethal West. Nile virus infection in aged mice. J. Virol..

[9] Domingo C., Lamerz J., Cadar D., Stojkovic M., Eisermann P., Merle U., Nitsche A., Schnitzler P. (2020). Severe Multiorgan Failure Following Yellow Fever Vaccination. Vaccines.

[10] Park J., Lee H.-Y., Khai L.T., Thuy N.T.T., Le Quynh M., Jang Y.-S. (2020). Addition of Partial Envelope Domain II into Envelope Domain III of Dengue Virus Antigen Potentiates the Induction of Virus-Neutralizing Antibodies and Induces Protective Immunity. Vaccines.

[11] Tremblay N., Freppel W., Sow A.A., Chatel-Chaix L. (2019). The Interplay between Dengue Virus and the Human Innate Immune System: A Game of Hide and Seek. Vaccines.

[12] Züst R., Dong H., Li X.-F., Chang D.C., Zhang B., Balakrishnan T., Toh Y.-X., Jiang T., Li S.-H., Deng Y.-Q. (2013). Rational Design of a Live Attenuated Dengue Vaccine: 2′-O-Methyltransferase Mutants Are Highly Attenuated and Immunogenic in Mice and Macaques. PLoS Pathog..

[13] Li S.-H., Dong H., Li X.-F., Xie X., Zhao H., Deng Y.-Q., Wang X.-Y., Ye Q., Zhu S.-Y., Wang H.-J. (2013). Rational Design of a Flavivirus Vaccine by Abolishing Viral RNA 2′-O Methylation. J. Virol..

[14] Muller D.A., Depelsenaire A.C.I., Shannon A., Watterson D., Corrie S.R., Owens N.S., Agyei-Yeboah C., Cheung S.T.M., Zhang J., Fernando G.J.P. (2019). Efficient Delivery of Dengue Virus Subunit Vaccines to the Skin by Microprojection Arrays. Vaccines.

[15] Gatherer D., Kohl A. (2016). Zika virus: A previously slow pandemic spreads rapidly through the Americas. J. Gen. Virol..

[16] Modjarrad K., Lin L., George S.L., Stephenson K.E., Eckels K.H., De La Barrera R., Jarman R.G., Sondergaard E., Tennant J., Ansel J.L. (2018). Preliminary aggregate safety and immunogenicity results from three trials of a purified inactivated Zika virus vaccine candidate: Phase 1, randomised, double-blind, placebo-controlled clinical trials. Lancet.

[17] Gaudinski M.R., Houser K.V., Morabito K.M., Hu Z., Yamshchikov G., Rothwell R.S., Berkowitz N., Mendoza F., Saunders J.G., Novik L. (2018). Safety, tolerability, and immunogenicity of two Zika virus DNA vaccine candidates in healthy adults: Randomised, open-label, phase 1 clinical trials. Lancet.

[18] Tebas P., Roberts C., Muthumani K., Reuschel E.L., Kudchodkar S.B., Zaidi F.I., White S., Khan A.S., Racine T., Choi H. (2017). Safety and Immunogenicity of an Anti–Zika Virus DNA Vaccine—Preliminary Report. N. Engl. J. Med..

[19] Frumence E., Viranaïcken W., Bos S., Alvarez-Martinez M.-T., Roche M., Arnaud J.-D., Gadéa G., Desprès P., Arnaud D.J. (2019). A Chimeric Zika Virus between Viral Strains MR766 and BeH819015 Highlights a Role for E-glycan Loop in Antibody-mediated Virus Neutralization. Vaccines.

[20] Ngono A.E., Vizcarra E.A., Tang W.W., Sheets N., Joo Y., Kim K., Gorman M.J., Diamond M.S., Shresta S. (2017). Mapping and Role of the CD8+ T Cell Response During Primary Zika Virus Infection in Mice. Cell Host Microbe.

[21] Hassert M., Harris M.G., Brien J.D., Pinto A.K. (2019). Identification of Protective CD8 T Cell Responses in a Mouse Model of Zika Virus Infection. Front. Immunol..

[22] Hassert M., Wolf K.J., Schwetye K.E., DiPaolo R.J., Brien J.D., Pinto A.K. (2018). CD4+T cells mediate protection against Zika associated severe disease in a mouse model of infection. PLoS Pathog..

[23] Garg H., Sedano M., Plata G., Punke E.B., Joshi A. (2017). Development of Virus-Like-Particle Vaccine and Reporter Assay for Zika Virus. J. Virol..

[24] Boigard H., Alimova A., Martin G.R., Katz A., Gottlieb P., Galarza J.M. (2017). Zika virus-like particle (VLP) based vaccine. PLoS Negl. Trop. Dis..

[25] Collins N.D., Shan C., Nunes B.T.D., Widen S.G., Shi P.-Y., Barrett A., Sarathy V.V. (2020). Using Next Generation Sequencing to Study the Genetic Diversity of Candidate Live Attenuated Zika Vaccines. Vaccines.

[26] Sharma P.K., Dmitriev I.P., Kashentseva E.A., Raes G., Li L., Kim S.W., Lu Z.-H., Arbeit J.M., Fleming T.P., Kaliberov S.A. (2018). Development of an adenovirus vector vaccine platform for targeting dendritic cells. Cancer Gene Ther..

[27] Steffen T.L., Hassert M., Hoft S.G., Stone E.T., Zhang J., Geerling E., Grimberg B.T., Roberts M.S., Pinto A.K., Brien J.D. (2020). Immunogenicity and Efficacy of a Recombinant Human Adenovirus Type 5 Vaccine against Zika Virus. Vaccines.

[28] Wang Y., Marin-Lopez A., Jiang J., Ledizet M., Fikrig E. (2020). Vaccination with Aedes aegypti AgBR1 Delays Lethal Mosquito-Borne Zika Virus Infection in Mice. Vaccines.

[29] Uraki R., Hastings A.K., Marin-Lopez A., Sumida T.S., Takahashi T., Grover J.R., Iwasaki A., Hafler D.A., Montgomery R.R., Fikrig E. (2019). Aedes aegypti AgBR1 antibodies modulate early Zika virus infection of mice. Nat. Microbiol..

[30] Frumence E., Viranaïcken W., Gadéa G., Desprès P. (2019). A GFP Reporter MR766-Based Flow Cytometry Neutralization Test for Rapid Detection of Zika Virus-Neutralizing Antibodies in Serum Specimens. Vaccines.

[31] Salat J., Mikulasek K., Larralde O., Formanová P., Chrdle A., Haviernik J., Elsterova J., Teislerova D., Palus M., Eyer L. (2020). Tick-Borne Encephalitis Virus Vaccines Contain Non-Structural Protein 1 Antigen and may Elicit NS1-Specific Antibody Responses in Vaccinated Individuals. Vaccines.

